# Understanding the impact of network structure on air travel pattern at different scales

**DOI:** 10.1371/journal.pone.0299897

**Published:** 2024-03-08

**Authors:** Hoai Nguyen Huynh, Kuan Luen Ng, Roy Toh, Ling Feng

**Affiliations:** 1 Institute of High Performance Computing (IHPC), Agency for Science, Technology and Research (A*STAR), Singapore, Republic of Singapore; 2 Changi Airport International Pte. Ltd. (CAI), Singapore, Republic of Singapore; East China Normal University, CHINA

## Abstract

This study examines the global air travel demand pattern using complex network analysis. Using the data for the top 50 airports based on passenger volume rankings, we investigate the relationship between network measures of nodes (airports) in the global flight network and their passenger volume. The analysis explores the network measures at various spatial scales, from individual airports to metropolitan areas and countries. Different attributes, such as flight route length and the number of airlines, are considered in the analysis. Certain attributes are found to be more relevant than others, and specific network measure models are found to better capture the dynamics of global air travel demand than others. Among the models, PageRank is found to be the most correlated with total passenger volume. Moreover, distance-based measures perform worse than the ones emphasising the number of airlines, particularly those counting the number of airlines operating a route, including codeshare. Using the PageRank score weighted by the number of airlines, we find that airports in Asian cities tend to have more traffic than expected, while European and North American airports have the potential to attract more passenger volume given their connectivity pattern. Additionally, we combine the network measures with socio-economic variables such as population and GDP to show that the network measures could greatly augment the traditional approaches to modelling and predicting air travel demand. We’ll also briefly discuss the implications of the findings in this study for airport planning and airline industry strategy.

## Introduction

Air travel has become an essential mode of transportation in modern society, connecting people and businesses across the world. As a result, it has become an integral part of modern society, playing a vital role in economic development, tourism, and global connectivity [1]. The aviation industry has experienced exponential growth in recent decades, with the International Air Transport Association (IATA) reporting that global air passenger traffic increased by 6.9% in 2018, reaching a total of 4.4 billion passengers [2]. This significant increase in air travel has resulted in a complex and dynamic global flight network that connects thousands of airports and destinations around the world [3, 4]. Despite the impact of the COVID-19 pandemic, the aviation industry is expected to recover and continue its exponential growth in recent years, with the number of passengers projected to reach 7.8 billion by 2040 (IATA June 2022 forecast) [5]. As air travel demand continues to increase, understanding the underlying structure and dynamics of the global air travel network is becoming increasingly important.

The spatial distribution of airports and their connectivity pattern can be studied as networks, with each airport representing a node and flight routes among them representing edges. The analysis of airport networks has been applied in various fields, including transportation [6], urban planning [7], and economics [8], to better understand the spatial structure of the transportation system and its impacts on the economy and society [9–11]. Moreover, the complex structure of the global flight network and its interaction with other factors, such as socio-economic variables, has made it an interesting subject for research using complex network analysis [12]. The analysis of air travel patterns as complex networks can provide insights into the structure and dynamics of the global air travel industry and can help airlines and airports make informed decisions about planning and strategy.

In recent years, there has been increasing interest in using complex network analysis to study airport networks and air travel patterns e.g. [13–17], with a particular focus on identifying key airports and routes, analysing their topological properties [18], and exploring the dynamics of the network over time [12]. These studies have investigated various network measures such as centrality, clustering, and community detection to identify important airports and flight routes in the global air travel network. In particular, Cheung et al. [17] investigated the community structure of the global airport network and identified key hubs and subnetworks. Guimerà et al. [19] used network analysis to identify the most important airports in the world, based on their centrality measures. On the other hand, several studies have also analysed the global air travel network using metrics such as degree centrality, betweenness centrality, and PageRank algorithm [20–23]. These studies have provided insights into the connectivity and centrality of different airports and regions in the global air travel network. For instance, Grubesic et al. [3] found that the global air travel network has a hierarchical structure, with a few dominant airports playing a crucial role in connecting different regions of the world. On the other hand, centrality measures could be used to assess the air travel network’s robustness and vulnerability to targeted attacks, such as the restructuring of the U.S. domestic air transportation network following the 9/11 attacks in order to improve the network’s efficiency and security [24].

In addition to analysing the structure of the air travel network, other studies have also explored the use of socio-economic variables to forecast air travel demand, e.g. [25, 26]. Several studies have found that socio-economic factors such as population, gross domestic product (GDP), and tourism are among the most significant predictors of passenger volume [27, 28]. Most of such studies have employed various statistical methods, such as regression analysis, time-series analysis, and spatial analysis, to identify the most important determinants of air travel demand [29]. For example, Addepalli et al. [30] explored the influential push and pull factors within social, demographic, and economic domains, highlighting their influence in the context of changing global geo-economic and political landscapes. Using multiple regression models, GDP, economic decision-making power, tourism functions, and distance from major air markets have been found account for over two-thirds of the variation in air service in European metropolitan areas [27]. Besides income and population level, results from the US highlight the significance of socio-economic mobility in predicting air travel demand and its importance in forecasting, demand management, and infrastructure planning [31]. Within the APEC region, air passenger flows data has been examined with both parametric linear models and non-parametric regression tree models to analyze the connections between passenger flows and various factors such as distances, populations, gross domestic products, per capita incomes, and employment rates [32]. In the Asia-Pacific and Latin America-Caribbean regions, typical economic drivers such as domestic own price and domestic income together with exogenous factors such as the global crude oil price have been used in the global vector autoregressive (GVAR) framework for air passenger forecasting [33]. In emerging economies such as Nigeria, it has been reported that that (GDP) and manufacturing production are significant factors influencing air travel in both the short- and long-term [34].

Besides stastical methods, a number of studies have employed other techniques such as gravity model [35–37] as inspired by Newton’s gravitational law or neural networks [38] from machine-learning. The gravity models are employed to approximate the amount of traffic flow between a pair of origin and destination as a function of their characteristics and the geographical distance between them. In such models, socio-economic measures of GDP and population of the origin and destination are often used to represents the amount of attraction that could be responsible for the amount of traffic flow between them [35, 36]. A wide range of characteristics, from the connectivity of the airports to environmental factors such as temperature or humidity [37], can also be considered in the model to predict the air passenger flows at different temporal scales. As an emerging area, AI-based techniques have also been applied to the problem of forecasting air passenger demand. It has been reported that machine-learning algorithms such as back-propagation neural networks can achieve high forecasting accuracy for air passenger demand [38]. However, such techniques are often cricised to lack explanability of the mechanisms behind the forecast, and therefore, posing barriers to practical applications by the stakeholders [33].

While the use of socio-economic variables to predict air travel demand has been extensively studied [39], there has been relatively little research on the combination of these variables with network analysis. Several studies have demonstrated that integrating the insights from network analysis with the predictive power of socio-economic variables can potentially improve the accuracy of air travel demand forecasting models [40, 41]. Although several attributes have been examined to analyse the structure of the air travel network, such as flight distance or the number of airlines operating a route [19], the influence of these attributes on passengers’ air travel behaviour remains poorly understood. As a result, there is a need to further explore the relationship between air travel network structure and air travel patterns to identify which aspect of the air travel network is more influential in determining passengers’ air travel behaviour. On the other hand, understanding how passengers behave in response to different network structures can also inform the design of more efficient and sustainable air transportation systems.

Moreover, despite the growing body of literature on air travel patterns, there has been limited research on the use of network analysis to understand the spatial scale of nodes in the air travel network. Specifically, few studies have examined the relationship between network measures and air travel demand at different spatial scales, from individual airports to metropolitan areas and countries. This gap presents an opportunity to better understand the dynamics of air travel demand at different spatial scales and the role of network measures in improving prediction accuracy. Examination of the air travel network at multiple spatial scales would allow us to gain a more comprehensive understanding of the structure and dynamics of the network and identify the factors that drive air travel demand at different levels.

In that context, this study aims to address the following research question: How does the structure of the global flight network influence the air travel patterns, and how well do the measures of such network structure predict the air travel demand? The study will examine the structure of the global flight network, with multiple attributes, and its relationship with air travel demand at different spatial scales, from individual airports to metropolitan areas and countries. Specifically, we will first consider different attributes such as flight route length and the number of airlines, both with and without codeshare agreements, to identify the network measures that are most relevant for predicting passenger volume. Secondly, we will combine network measures with socio-economic variables to investigate the quality of passenger volume prediction with and without network measures at both the metropolitan area and country level. By doing so, we aim to shed light on the relationship between network measures and air travel demand at different spatial scales, providing insights into the factors that drive air travel patterns and demand. We will also discuss the potential implications of the findings for airport planning and airline industry strategy.

## Data and methods

### Data

The data employed in this study can be grouped into 5 different categories, namely the location of airports, flight routes among them, population of the area corresponding to the airports, economic measure of such areas, and the total passenger volume of the individual airports.

#### Airports and flight routes

The data on airports and flight routes among them were obtained from two open databases, namely OurAirports [42] and OpenFlights [43]. For the airports, we have the information of their name, trigram IATA code, and location, specifying the exact spatial coordinates, the city and country that they belong to. This information will be used for analysis at both the individual airport (metropolitan area) and country level (see also Sec Population and economic indicator). For the flight routes, we have the information on the airlines providing the service between a pair of airports, with codeshare included. Codeshare is a common aviation practice in which two or more airlines share the same flight by allowing passengers to book tickets on one airline’s flight, which is operated by another airline. Using the exact spatial coordinates of the airports, we also derive the geodesic distance between a pair of airports as the proxy of their flight route length.

Within the scope of this analysis, we only consider the commercial flights and the corresponding airports. Subject to the availability of data, we only study the flight routes that operated in 2014. While we are aware that this choice may not necessarily reflect the most recent state of the global air network, we believe that outcomes of the analysis should not be significantly altered upon inspection of the airports’ total passenger volume (at least until before the COVID-19 pandemic, see Sec Passenger volume below). Furthermore, it should be emphasised that the focus of this study is the relationship between the global flight network structure and the air travel demand. Therefore, as long as the corresponding datasets are temporally consistent, we argue that the results are practically valid and relevant in the present context.

#### Passenger volume

In this study, we choose to analyse the top 50 airports (see Table 1) based on the 2014 total passenger volume (including both enplaning and deplaning passengers) reported by the Airports Council International (ACI) [44]. Together, these 50 airports account for about 36% of worldwide air passengers in 2014. The list of 50 busiest airports by passenger traffic has been largely consistent with a steady rate of traffic increment among the airports and minimal changes in their rankings between 2014 and 2019 (before the COVID-19 pandemic), with 92% (46/50) of the airports that were in the top 50 in 2014 remaining so in 2019. For this reason, we believe that an analysis that focuses on this group of airports would yield similar results between 2014 and 2019, and that the results, although for 2014, are still highly relevant to the recent time.

**Table 1:** List of 50 busiest airports by passenger traffic in 2014.

IATA	Metropolis	Country		IATA	Metropolis	Country
SYD	Sydney	Australia		ICN	Seoul-Incheon	South Korea
GRU	Sao Paulo	Brazil		BCN	Barcelona	Spain
YYZ	Toronto	Canada		MAD	Madrid	
PEK	Beijing	China		TPE	Taoyuan	Taiwan
CTU	Chengdu			BKK	Bangkok	Thailand
CAN	Guangzhou			IST	Istanbul	Turkey
PVG	Shanghai			DXB	Dubai	UAE
SHA				LHR	London	UK
SZX	Shenzhen			LGW		
CDG	Paris	France		ATL	Atlanta	USA
FRA	Frankfurt am Main	Germany		CLT	Charlotte	
MUC	Munich			ORD	Chicago	
HKG	Hong Kong	Hong Kong		DFW	Dallas	
DEL	Delhi	India		DEN	Denver	
BOM	Mumbai			IAH	Houston	
CGK	Jakarta	Indonesia		LAS	Las Vegas	
FCO	Rome	Italy		LAX	Los Angeles	
HND	Tokyo	Japan		MIA	Miami	
NRT				MSP	Minneapolis	
KUL	Kuala Lumpur	Malaysia		JFK	New York	
MEX	Mexico City	Mexico		EWR		
AMS	Rotterdam-Amsterdam	Netherlands		MCO	Orlando	
MNL	Manila	Philippines		PHX	Phoenix	
DME	Moscow	Russia		SFO	San Francisco	
SIN	Singapore	Singapore		SEA	Seattle	

#### Population and economic indicator

**Fig 1:**
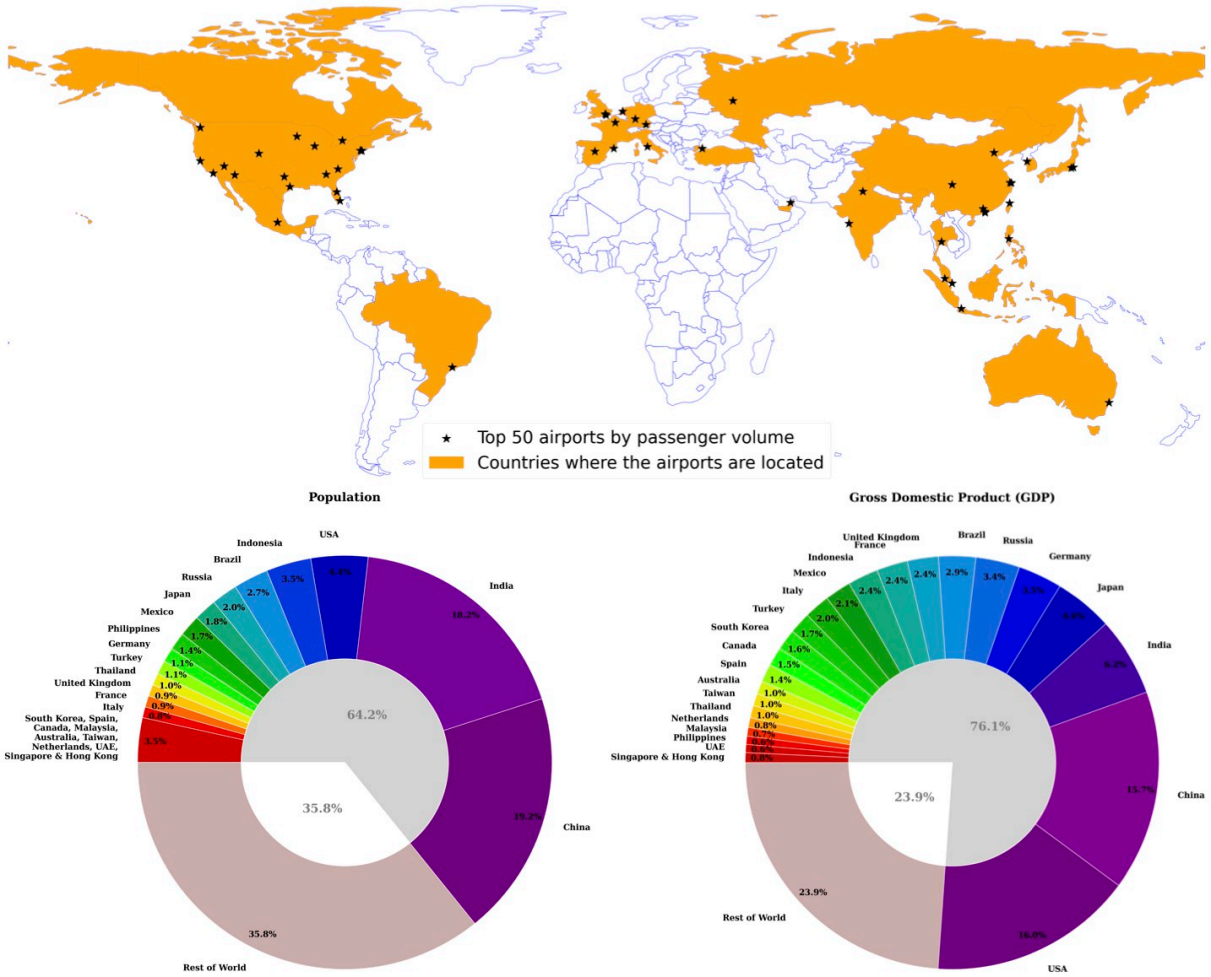
Top 50 busiest airports by passenger traffic in 2014 and their respective countries. The pie charts show the countries’ population (left) and GDP (right) as fraction of the entire world. The world map showing countries’ boundary was generated using GeoPandas [47].

Apart from the global flight network and passenger volume at individual airports, we also incorporate socio-economic variables of population and economic output in the analysis. As mentioned earlier, we analyse the data at two levels, the metropolitan area (corresponding to the individual airports) and country level. The data was obtained from various public sources, including the International Monetary Fund [45] and the Brookings Institution [46], both at the metropolitan and country level. For the economic indicator, we use the gross domestic product (GDP) at the country level, and the gross metropolitan product (GMP) at the metropolitan level. To be consistent with the flight network data, the population and GDP/GMP data was also obtained for the year 2014. The select 50 airports are located in 46 different metropolitan areas in 25 countries (see Fig 1) across 4 continents. These 25 countries accounted for almost two thirds (64.2%) of the world’s population and more than three quarters (76.1%) of the global GDP in 2014.

### Global flight network

#### Network construction

Using the data on airports and flight routes described in Sec Airports and flight routes, we construct a network of airports with the nodes representing the individual airports, and a pair of airports are connected by a link if there is a flight between them. Because of the nature of the flight from an origin to a destination airport, the constructed network is inherently a directed network. Furthermore, pre-processing of the data is also performed to ensure that the constructed network is a connected one with a path existing between any pair of nodes, i.e. every node is reachable from all other nodes. Overall, we have in total 3,360 airports and 37,461 flight links between them in this network. It should be noted that we construct a full global network of all airports for the analysis of network measures and subsequently select the 50 airports listed in Table 1 for the analysis of passenger volume.

#### Link attribute

**Fig 2:**
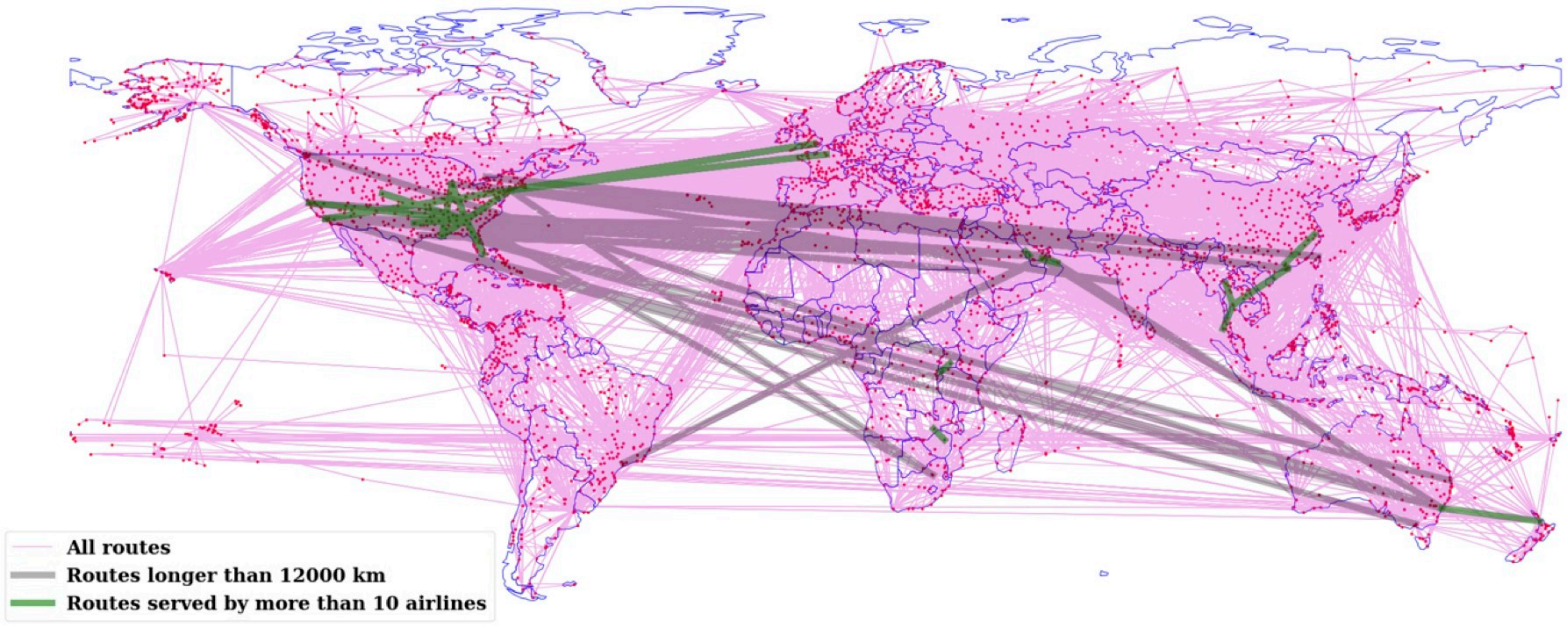
Global flight network showing the commercial flight routes among the airports. The links with the number of airlines *W*_*A*_ > 10 and distance *W*_*D*_ > 12, 000 km are highlighted to demonstrated the attributes of the links. The world map showing countries’ boundary was generated using GeoPandas [47].

For the flight links, or network edges, we compute 7 attributes that account for the geodesic distance and the number of airlines serving the route, including both with and without codeshare. We denote these 7 attributes as *W*_*A*_, *W*_*A*(*NCS*)_, *W*_*D*_, *W*_*C*_, *W*_*C*(*NCS*)_, *W*_*F*_ and *W*_*F*(*NCS*)_, respectively (see their summary in Table 2). *W*_*A*_ simply counts the number of airlines serving a route between two airports, without considering whether an airline operates the route as a codeshare with another airline. *W*_*A*(*NCS*)_, on the other hand, counts the number of airlines serving the route, excluding the code-sharing ones (“NCS” stands for “no codeshare”), i.e. only counting the operating carriers. *W*_*D*_ is simply the geodesic distance between two airports. The composite *W*_*C*_ (and the no codeshare version *W*_*C*(*NCS*)_), which is the ratio between *W*_*A*_ (or *W*_*A*(*NCS*)_) and *W*_*D*_, represents the closeness between two airports, in the sense that more airlines operating the route or shorter distance would indicate a closer relationship between them. Conversely, *W*_*F*_ (and *W*_*F*(*NCS*)_), which is the ratio between *W*_*D*_ and *W*_*A*_ (or *W*_*A*(*NCS*)_), represents the farness between the two airports, in the same interpretation. A visualisation of the global flight network with highlights of *W*_*A*_ and *W*_*D*_ is shown in Fig 2.

**Table 2:** Description of the 7 attributes of the links in the global flight network.

Symbol	Attribute name	Description
*W*_*A*_	Number of airlines (with codeshare)	The number of airlines serving a route between a pair of airports, including airlines in code-sharing arrangements
*W*_*A*(*NCS*)_	Number of airlines (without codeshare)	The number of airlines serving a route between a pair of airports, without codeshare, i.e. considering only the operating ones
*W*_*D*_	Flight route distance	The geodesic distance between two airports
*W*_*C*_	Closeness (with codeshare)	The ratio *W*_*A*_/*W*_*D*_, with more airlines operating the route or shorter distance between a pair of airports indicating a closer relationship between them
*W*_*C*(*NCS*)_	Closeness (without codeshare)	The ratio *W*_*A*(*NCS*)_/*W*_*D*_, with the same description as *W*_*C*_ but without codeshare
*W*_*F*_	Farness (with codeshare)	The ratio *W*_*D*_/*W*_*A*_ (the reverse of *W*_*C*_), with less airlines operating the route or longer distance between 2 airports indicating a weaker tie between them
*W*_*F*(*NCS*)_	Farness (without codeshare)	The ratio *W*_*D*_/*W*_*A*(*NCS*)_ (the reverse of *W*_*C*(*NCS*)_), with the same description as *W*_*F*_ but without codeshare

#### Network measures

After constructing the global flight network, we analyse it using different models of the network measures. These models include the 3 most commonly used network measures in the literature, namely betweenness centrality (for both nodes and edges), closeness centrality, and degree centrality. Among these measures, node betweenness centrality measures how often a node in the network lies on the shortest path between all pairs of other nodes. Similarly, edge betweenness centrality works the same way but applying to a route connecting two airports, instead of an airport itself. But given the focus of this study is on the airports, we only consider node betweenness centrality in the subsequent analysis. On the other hand, closeness centrality measures how close a node is to the centre of the network. The degree centrality counts the number of connections that a node has with others in the network.

Besides these network measures, eigenvector centrality is also often used. In this study, we will employ a variant of the eigenvector centrality, called PageRank [48], which is based on a mathematical model of random walk, or random exploration of the network, to model the interactions among the nodes. The interaction model of PageRank computes the (relative) importance of the nodes in the network by taking into account the quality of connections that each node has. Mathematically, the PageRank score of a node (airport) a in the network with N nodes is given by

$$\begin{array}{c} x\left(a\right)=\frac{1-\beta }{N}+\beta \sum_{i}^{k}\frac{x(\nu_{i}^{\left(a\right)})}{k(\nu_{i}^{\left(a\right)})} \end{array}$$ (1)

in which *k*(*a*) is the number of connections that node *a* has, *x*(*a*) the PageRank score of *a*, $\nu_{i}^{\left(a\right)}$ the *i*-th neighbour of *a*, and *β* is a damping factor that is typically taken to be 0.85 [48].

### Passenger volume prediction

To examine the impact of the flight network structure on passenger traffic, we construct different regression models to see how well the addition of network measure to the tradition approach of GDP and population could predict the air travel demand. Specifically, we employ the Ordinary Least Square (OLS) method to fit the data, through minimising the sum of squared differences between the observed and predicted values. Taking total passenger volume as the dependent variable, we will have models for different combinations of the independent variables, namely GDP, population, and network measure. Typically, we will have base models with the inclusion of each of the individual independent variables. Subsequently, the variables of GDP and population will be considered concurrently in another model, as well as together with the network measure in the full model. As a standard procedure for OLS models, we perform the test for multicollinearity with Variance Inflation Factors (VIF) and use the value of VIF less than 5 as a good indicator for low correlation among the predictors [49]. The analysis will be performed at both the metropolitan area and country level to understand the role of the spatial scale in the networked relationship among different places and reveal the one that would best capture the dynamics of air travel flow.

## Results

### Correlation between network measures and passenger volume

#### Details of the 19 network measures

Based on the 4 models of network measures described in Sec Network measures together with the attributes associated with the flight routes (see Sec Link attribute), we computed a host of different network measures (for the full network of 3,360 airports). The simplest in the lot, the degree centrality DC of an airport simly counts the number of other airports that have direct flights to and from, i.e. the number of (direct) connections that the airport has with others. The group of network measures based on the PageRank model contains 5 variants. The unweighted PageRank score treats every edge in the global flight network equally and computes the score of each node according to Eq 1. The other variants in this group further consider weightage of the routes between the airport using the number of airlines serving the routes as well as the ratio between the number of airlines and the route distance (see Table 2 for a summary of the weightage attributes). The group of network measures based on the betweenness model contains 4 variants. These measures give higher score to the airports that are more frequently included in the shortest (based on the weightage of interest) chain of routes between different pairs of origin and destination airports across the world. Similarly, the group of measures based on the closeness model consider the shortest chain of routes between the airport of interest and all other airports. Finally, we consider a group of network measures that are the product of PageRank score and betweenness centrality.

In total, we have 19 different network measures (see Table 3), and we can compute the correlation between these measures with the total passenger volume for the selected top 50 airports (see Sec Passenger volume). For each measure *m*, the correlation is computed as the Pearson correlation coefficient *r*_*m*_, which is given by

$$\begin{array}{c} r_{m}=\frac{n\sum_{a}m_{a}V_{a}-\sum_{a}m_{a}\sum_{a}V_{a}}{\sqrt{n\sum_{a}m_{a}^{2}-{\left(\sum_{a}m_{a}\right)}^{2}}\sqrt{n\sum_{a}V_{a}^{2}-{\left(\sum_{a}V_{a}\right)}^{2}}} \end{array}$$ (2)

in which *n* = 50 is the number of airports considered in the analysis, *m*_*a*_ the corresponding network measure for the airport *a*, and *V*_*a*_ is the total passenger volume of the airport *a*.

**Table 3:** Description of the 19 network measures based on the different models. Refer to Sec Link attribute for the details of weights.

Name	Description	Remarks	Model
DC	Degree centrality	Number of other airports having flights to/from	Degree
PR	PageRank score	Every edge treated equally	PageRank
PR_A_	Weighted PageRank score	Weighted by *W*_*A*_ (number of airlines)	
PR_A(NCS)_		Weighted by *W*_*A*(*NCS*)_ (excluding codeshare)	
PR_C_		Weighted by *W*_*C*_ = *W*_*A*_/*W*_*D*_	
PR_C(NCS)_		Weighted by *W*_*C*(*NCS*)_	
BC	Betweenness centrality	Every edge treated equally	Betweenness
BC_D_	Weighted betweenness centrality	Weighted by *W*_*D*_ (flight route distance)	
BC_F_		Weighted by *W*_*F*_ = *W*_*D*_/*W*_*A*_	
BC_F(NCS)_		Weighted by *W*_*F*(*NCS*)_	
CC	Closeness centrality	Every edge treated equally	Closeness
CC_D_	Weighted closeness centrality	Weighted by *W*_*D*_	
CC_F_		Weighted by *W*_*F*_	
CC_F(NCS)_		Weighted by *W*_*F*(*NCS*)_	
BP	PageRank and betweenness	Product of PR and BC	Betweenness and PageRank
BC_AD_	Weighted PageRank and betweenness centrality	Product of PR_A_ and BC_D_	
BC_AD(NCS)_		Product of PR_A(NCS)_ and BC_D(NCS)_	
BC_CF_		Product of PR_C_ and BC_F_	
BC_CF(NCS)_		Product of PR_C(NCS)_ and BC_F(NCS)_	

#### Correlation between the network measures and passenger volume

**Fig 3:**
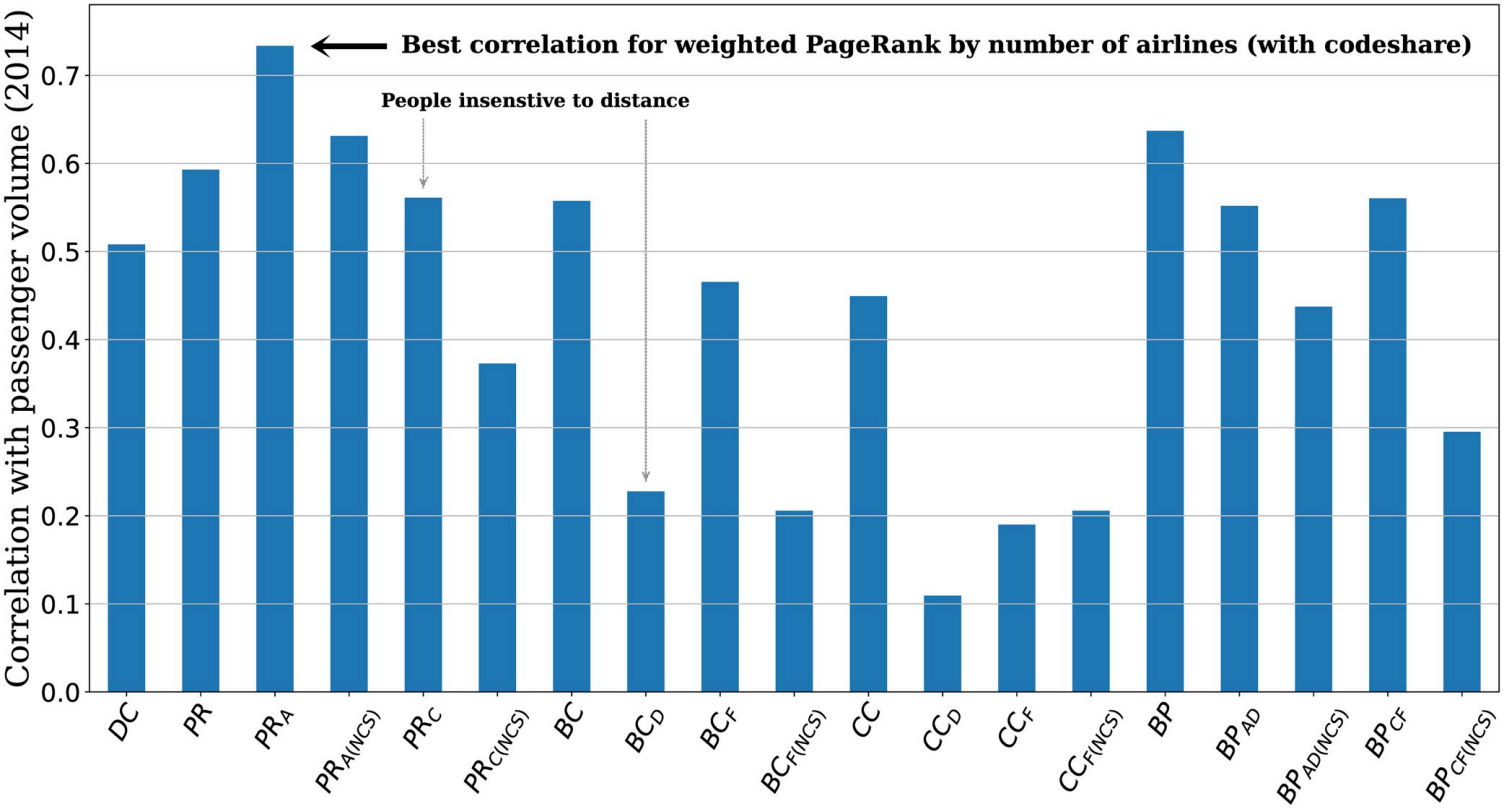
Correlation between different network measures and passenger volume for the 50 airports considered in this study. Measures (such as PR_C_ or BC_D_) that take the flight route distance into account perform worse than the unweighted ones (PR or BC) or those weighted by the number of airlines serving the route (PR_A_ or BC_F_), suggesting insensitivity to distance in air travelling. Refer to Table 3 for the description of the network measures.

It could be observed that the network measure of PageRank weighted by the number of airlines (PR_A_) appears to be best correlated with the passenger volume (see Fig 3) with a coefficient of about 0.73. Other network measures based on the PageRank model also show considerable amount of correlation of more than 0.5 (except PR_C(NCS)_) but less than 0.7. The degree centrality, which simply counts the number of connections that an airport has with others, shows an average amount of correlation of about 0.5 and generally performs poorer than the PageRank model. On the other hand, the betweenness or closeness centrality measures are poorly correlated with the passenger volume, especially when the weightage is involved. The versions of these models that perform the best are the unweighted ones, which treat every connection equally. The combined model of betweenness centrality and PageRank appear to work better than the betweenness model itself but not as good as the PageRank model alone.

The observed correlation pattern in Fig 3 suggests that the network measure models based on shortest path calculation such as betweenness and closeness would not agree well with the passenger traffic. At the same time, the models that place emphasis on the number of connections that a node has with others appear to capture the essential dynamics of the passenger flow within the airport network. It is also worth pointing out that network measures that take the flight route distance into account (e.g. PR_C_ or BC_D_) perform worse than the unweighted ones or those weighted by the number of airlines serving the route, suggesting insensitivity to distance in air travelling. Similarly, if we exclude the codeshare airlines from the calculation, the network measures would also perform worse.

#### Comparison between airports in different regions

**Fig 4:**
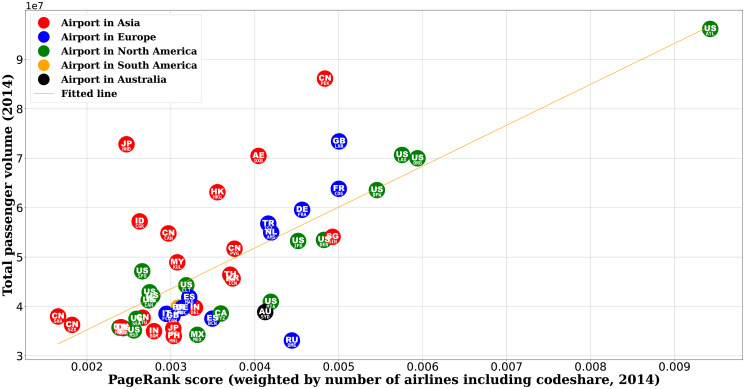
Comparison between total passenger volume and PageRank score weighted by the number of airlines for the 50 airports considered in this study. For each airport, its corresponding country is labelled by the two-letter country code as defined in ISO 3166-1.

After identifying the network measure of PageRank weighted by the number of airlines (PRA) that would best correlate with the passenger traffic, we can take a closer look at the comparison between the PageRank score and the passenger volume. If we colour code the airports by their respective country and region (see Fig 4), we can observe that quite a number of airports in Asia have the total passenger volume above the linear fitted line, suggesting they have more air travel demand than the network measure would predict. Conversely, most airports in Europe and North America have less air travel demand than predicted by the network measure.

**Fig 5:**
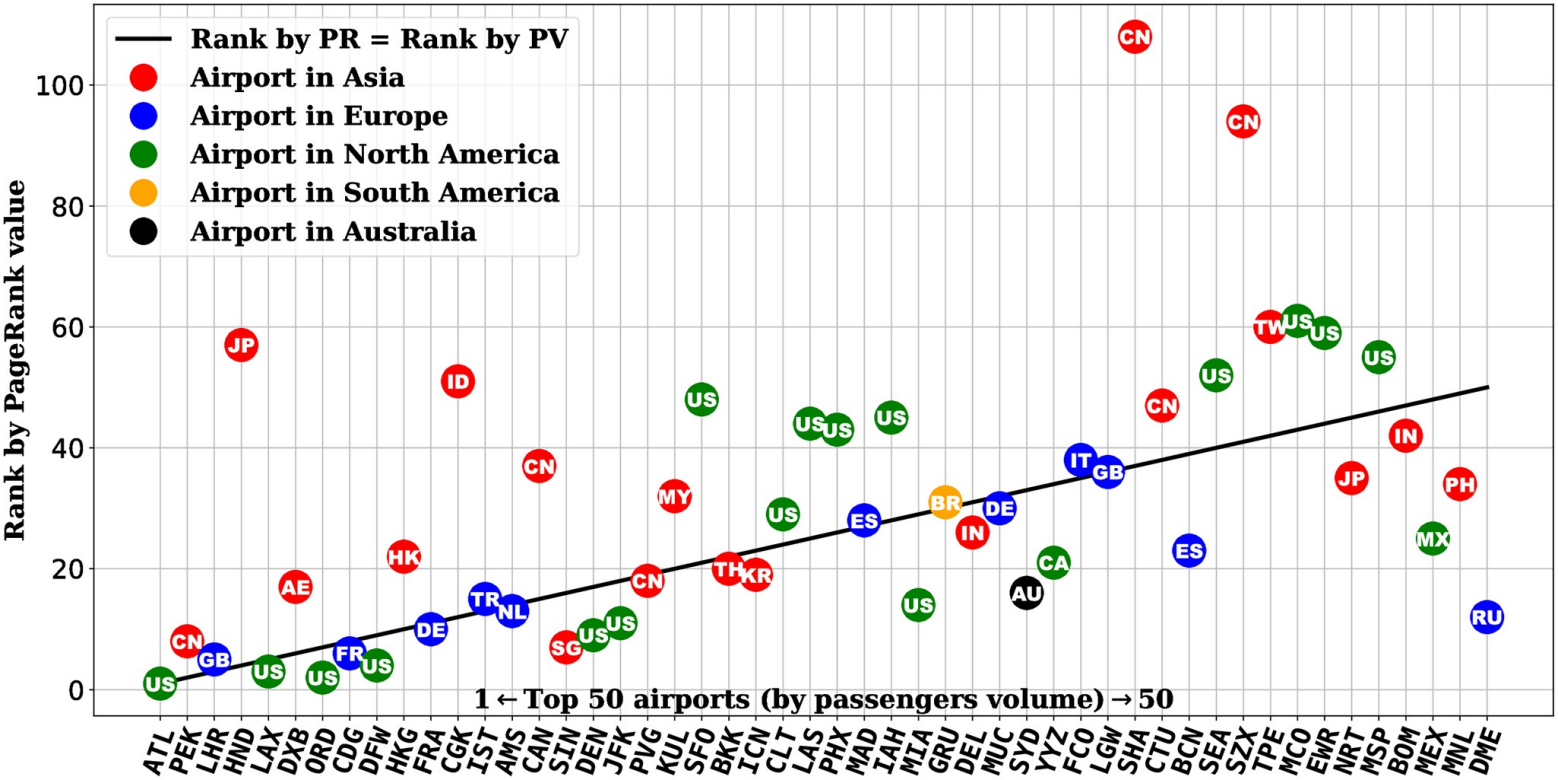
Comparison between ranking of airports by total passenger volume and PageRank score weighted by the number of airlines for the 50 airports considered in this study. For each airport, its corresponding country is labelled by the two-letter country code as defined in ISO 3166-1.

The pattern of airports by region observed in Fig 4 also agrees with a similar analysis using the integer rank value of the airports (see Fig 5) instead of the real value of the network score and passenger volume. The result may indicate that given the flight network connections or topology, the airports in Asia tend to have more travel demand than expected, whereas travel demand in Europe and North America is less than expected given the network structure as the baseline. This could be attributed to GDP and population, which we will look at next.

### Prediction of the passenger volume

Traditionally, the prediction of the passenger volume has been performed using trend extracted from the historical traffic data combined with macro socio-economic data such as population and GDP. On the other hand, airline networks have also been recognised as one of the key factors that influence global air travel demand. However, variables that account for the network structure based on a systemic characterisation, such as those offered by the network models being discussed here, have not been properly incorporated in the existing forecast models. In this section, we will discuss how the network measures described in Sec Details of the 19 network measures, in particular PR_A_, would contribute to the prediction of the passenger volume, besides variables such as population and GDP. We will construct different regression models with various combinations of independent variables, including GDP, population, and network measures, to predict the total passenger volume. This analysis will be conducted at both metropolitan and country levels, to understand the role of spatial scales in capturing the dynamics of air travel flow among different locations. This multi-level analysis would provide insights into the geographical scope and influence of the individual flight routes as well as the interdependence between international and domestic air traffic.

#### Regression at metropolitan level

At the metropolitan level, we use the data for the metropolis corresponding to the airports of interest. In particular, for every airport, we use the population and GMP of the metropolitan area that houses the airport together with its network score (PR_A_) to predict the total passenger volume of that airport (see Table 4).

**Table 4:** Regression models at metropolitan level. An “X” indicates the inclusion of the corresponding independent variable in the regression model.

Predictor for passenger volume			*R* ^2^
Gross metropolitan product	Population (metropolitan)	Network score (PR_A_ weighted PageRank by the number of airlines, including codeshare)	
X***			0.490
	X***		0.347
X*	X		0.490
		X***	0.678
X	X*	X***	0.798

*p < 0.05,

**p < 0.01,

***p < 0.001

We observe that GMP or population alone would poorly predict the total passenger volume for the airport located in the respective metropolitan area, with *R*^2^ value being less than 0.5. Combination of the two yields similar value of *R*^2^ less than 0.5. On the other hand, network measure proves to outperform the socio-economic variables in predicting the passenger traffic. In particular, PR_A_ alone can significantly improve the prediction, with *R*^2^ being significantly larger at more than 0.5. In the full model where we consider both the network measure and socio-economic variables, an improvement is observed when both GMP and population are added, with *R*^2^ being larger at almost 0.8. Yet, the p-value for both GMP and population again indicates their statistical insignificance. This suggests that network measure would be a significant predictor of the air travel demand at the metropolitan level.

#### Regression at country level

At the country level, we simply sum the value of network measure over the airports of the same country to represent the measure for the entire country. Similarly, we also sum the value of passenger volume of the same-country airports. However, we have two ways to compute the aggregate GDP and population at the country level. The first way is to perform a summation over the corresponding areas in which the airports of interest are located. For example, among the countries, Spain has 2 airports selected for this analysis, namely Madrid and Barcelona. As a result, the summed GDP for Spain would be the sum of GMP for Madrid and Barcelona metropolitan areas, and similarly for population. The second way is to use the official GDP and population for the whole country even though we only consider a few airports in that country. For example, the passenger volume for the United Kingdom is only from London’s Heathrow and Gatwick airports, but the GDP and population will be for the whole of UK. After that, we build the regression models for different combinations of the variables to predict the passenger traffic at the country level as described in Table 5 below.

**Table 5:** Regression models at country level. An “X” indicates the inclusion of the corresponding independent variable in the regression model.

Predictor for passenger volume					*R* ^2^
GDP (sum over the corresponding metropolitan areas)	Population (sum over the corresponding metropolitan areas)	GDP (whole country)	Population (whole country)	Network score (sum over the corresponding airports)	
X***					0.878
	X***				0.638
				X***	0.984
X***	X				0.879
X	X***			X***	0.994
		X***			0.722
			X		0.094
		X***	X***		0.883
		X***	X	X***	0.992

*p < 0.05,

**p < 0.01,

***p < 0.001

Generally, we observe that the GDP alone, both by summation of the metropolitan areas and whole country, would predict the passenger volume better than population alone. The corresponding variable as the summation of the metropolitan areas expectedly perform better than the whole country, especially in the case of population. The combination of GDP and population only shows a slight improvement in the case of whole country, even though the passenger volume is only for the selected airports, whereas the summed population exhibits unreliable fit. However, network measure alone can predict the passenger volume very well, even better than GDP and/or population (both summation of the metropolitan areas and whole country). Comparing with the corresponding results reported in Sec Regression at metropolitan level, we observe that this prediction is better at the country level, or aggregation of selected airports, than at the metropolitan level, or individual airports. And certainly, when combining network measure with GDP and population, we always observe the improvement in the prediction.

## Discussion

### PageRank works best among the network measures

Among a host of network measures that were developed based on different models, PageRank appears to outperform the rest in predicting the passenger traffic. This would suggest that the mathematical model underpinning the network measure basically captures the total flow through the airports and more importantly, the share of flow among the destinations of a particular airport, as reflected in the second term in Eq 1. Although, the PageRank model was not specifically designed for air travel demand prediction, we can do a mapping to interpret the terms in the equation that reflect the actual dynamics of the flow within the flight network, similar to the case of other urban transport network [50]. It can be argued that in this PageRank model, a node benefits more from having direct connection with an important node in the network.

As with any correlation analysis, the relationship between the PageRank score and passenger volume is not a causal one but it forms the basis for further investigation. Future studies could look into the mechanisms of how flight network structure could drive the passenger traffic at different nodes in the network. This could be studied, for example, through the evolution of the network structure with opening (and cessation) of new flight routes over time and the corresponding dynamics of passenger traffic. This type of analysis requires longitudinal data on both the flight routes and the airport-to-airport passenger volume over the years, which is not available in the current study.

**Fig 6:**
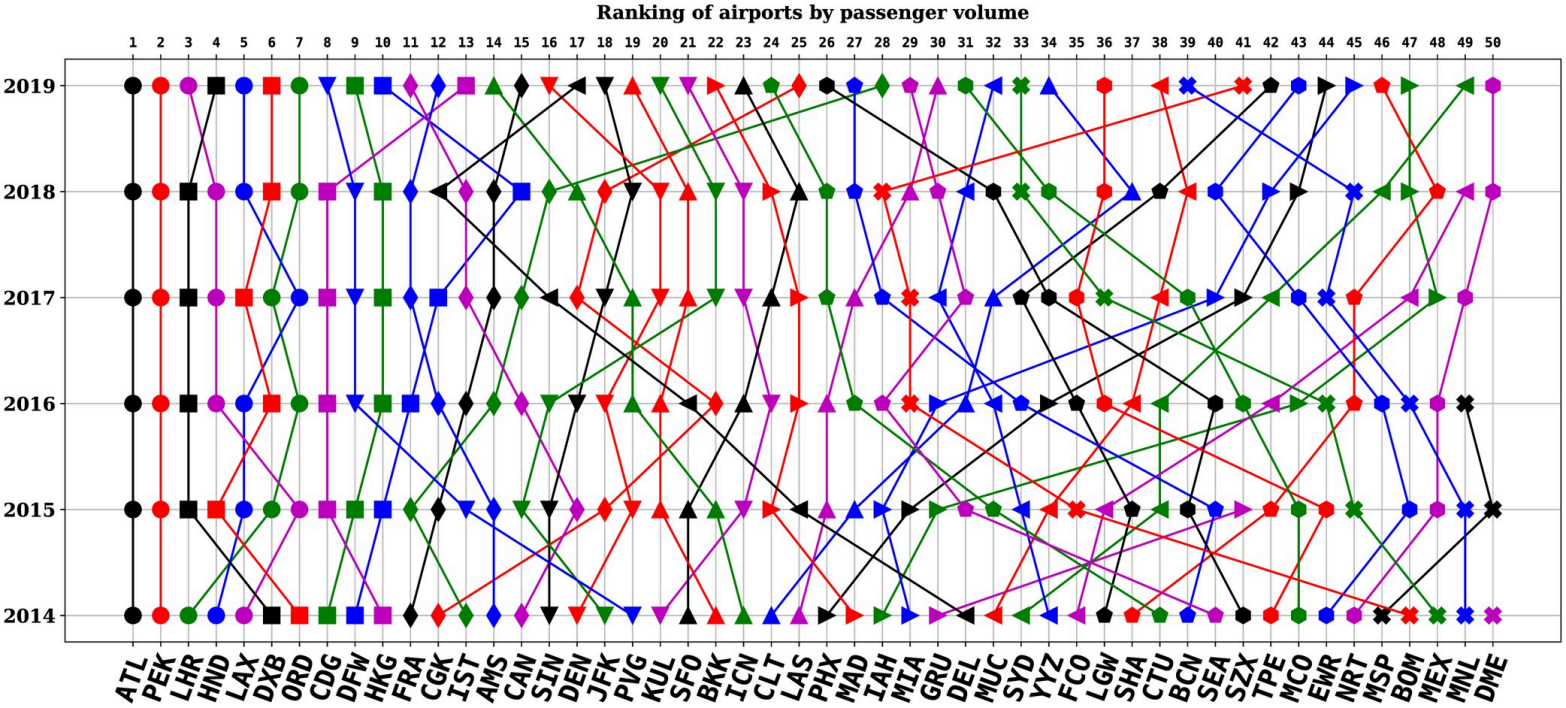
Ranking of the 50 selected airports by total passenger volume from 2014 to 2019. The lines show the movement of the airports by their ranking position. For example, Hartsfield-Jackson Atlanta International Airport (ATL) and Beijing Capital International Airport (PEK) were consistently ranked 1^st^ and 2^nd^ through the period. On the other hand, Soekarno–Hatta International Airport (CGK) were ranked 12^th^ in 2014, 18^th^ in 2015, 22^nd^ in 2016, 17^th^ in 2017, 18^th^ in 2018, and 25^th^ in 2019.

On the other hand, a more recent data set on flight network could also help to confirm the assumption made in this study that a corresponding analysis in 2019 (before the COVID-19 pandemic) would yield similar results obtained for 2014, given 46 of the top 50 airports in 2014 remained so in 2019. However, there were changes in ranking of the airports over the years (see Fig 6) and understanding their dynamics of passenger traffic with respect to network structure and socio-economic variables would gain further insights into their relationship.

### Distance vs. number of airlines

It should be noted that the measures that favour distance appear to perform worse than those that put emphasis on the number of airlines. In other words, passengers seem to be influenced more by the number of airlines that are available to serve a particular route more than the actual flight distance from the origin to the destination airport. The impact of the geographical distance of the flight route could be related to the fact that the number of air passengers could be the most significant for mid-range flights (world-regional scale) and less so for short-range flights (because of the availability and competition of other transport modes) and long-range flights that span the globe. It could also be observed in Fig 3 that measures incorporating both distance and the number of airlines also perform worse than those with the number of airlines only. On the one hand, this result might suggest that people are insensitive to distance, and they would travel provided they could get a ticket to their destination, without pondering the issue of distance travel. On the other hand, it might well be the case that more airlines providing the service would result in competitive air ticket prices, which in turn encourage more people to travel. However, testing the effect of passenger volume at different flight distance requires higher resolution of passenger volume data such as the airport-to-airport flow volume, which is not available in this study and will be examined in a separate study. Similarly, the airfare is not within the scope of the present analysis and will be explored in a future study.

### Codeshare

We can observe that if we exclude the codeshare airlines from the calculation, the network measures would perform worse. This applies to all relevant network measure models considered in this study, including betweenness, closeness and PageRank. This further highlights the role of the number of airlines serving a route that we already discussed above. One the one hand, it may be thought that including the code-sharing airlines that don’t physically operate the route may overestimate the travel demand. Yet, it could be argued that codeshare indeed reflects the actual demand of passenger because that is the actual number of air tickets sold which translates to the passenger volume on a particular route. However, this point should be scrutinised further with more detailed data on the actual flight operation of the airlines, such as the frequency of the flights or even the fleet size. With such data, we may examine whether code-sharing airlines operate more flights than others, which essentially drive the passenger traffic.

### Spatial scale: City vs. country

The analysis of the relationship between the network measure, together with the socio-economic variables, and the passenger volume at two different spatial scales of metropolitan area and country allows us to understand the impact of local, regional and global factors on the performance of individual airports and the countries’ air transport system. The results of the regression models in Tables 4 and 5 suggest that the passenger traffic at the country level could be better predicted than at the metropolitan area. This could indicate that while an airport typically serves a metropolitan area, it can also be the gateway to a country. For example, major international flights into the UK (especially the long haul from Asia/Australia, or even North America) would mostly be through London Heathrow. The passengers arriving in Heathrow may not necessary be bounded for the London region, but dispersed to others across the country, for which the national GDP and population would better reflect the travel demand.

The role of being the gateway to a country could also be extended to a local region within the country. For example, an airport in a metropolitan area in the US could serve as a local hub for the surrounding cities. In fact, most regions in the US would have a major airport designated as the local hub that collects the international arrivals before distributing them to domestic destinations for inbound trips, and vice versa to collect the domestics arrivals before transferring them to international destinations for outbound trips. This could explain why whole country could work better than the summation of the metropolitan areas corresponding the selected airports, when the surrounding cities could contribute to drive the passenger traffic but are not captured in the metropolitan areas’ socio-economic calculation.

On a technical note, the OLS regression models in Sec Prediction of the passenger volume were tested for multicollinearity using the Variance Inflation Factors (VIF). We use the value of VIF less than 5 as a good indicator for low correlation among the predictors [49]. In the case of regression models at the metropolitan area level, the VIF values for all the variables were less than 5. At the country level, the VIF values are higher but still within the acceptable range of less than 10 [51]. There might be concern that the OLS may not be ideal for network measures due to possibility of correlation among the variables. In future studies, we will explore other models such as Multiple Regression Quadratic Assignment Procedure (MRQAP) or Exponential Random Graph Model (ERGM) to confirm the findings in this study.

### Asian vs. European and North American airports

Using the network measure as a method of benchmarking, we observe that the airports in Asia tend to have more passenger traffic whereas the ones in Europe and North America tend to have less. In other words, given the air network connections, Asian airports are busier than expected. On the other hand, European and North American airports still have potential for growth if their route connectivity could be better exploited. This can also be clearly observed when we compare the ranking of the airports by their PageRank score and that by their passenger volume (see Fig 5). This could be attributed to the fact that Asian cities are among the most populous in the world, whose large population coupled with the booming local economy could be a key driving force for air travel. Conversely, the developed economies in Europe and North America could enjoy the benefit of having good infrastructure that results in a well-connected network for mobility, including air travel. However, the high level of transport infrastructure development for all modes of mobility could create a competition for passenger traffic that may draw demand from air travel and redistribute that to other modes of transport, especially on land, i.e. road and rail. Future studies could look into this interaction between different modes of transport subject to the availability of the corresponding data.

### Value of network analysis and practical implications

The analysis performed in this study demonstrates the value of network analysis as a powerful tool to better understand the dynamics of air travel demand by providing insights into the patterns of flight route connectivity and their relationship with flows of passenger traffic. The findings highlight the importance of evaluating airports’ performance from the perspective of their connectivity to other airports, rather than simply looking at their individual characteristics, including the associated socio-economic features such as population and GDP. As demonstrated in the regression models, the addition of network measures could greatly augment the existing approaches in projecting the future travel demand, beyond the projection of population and GDP growth. Furthermore, projecting the evolution of the flight network could also greatly improve the prediction of future air travel demand, which could help operators, including airports and airlines, identify and take advantage of new market opportunities as well as prepare for potential challenges.

The practical implications derived from this study highlight the need for a paradigm shift in evaluating airport performance and informing strategic decisions within the aviation industry. By incorporating network analysis, airports and aviation authorities can gain a more comprehensive understanding of air travel dynamics, focusing on the connectivity of airports rather than relying solely on demographic and economic metrics. Airlines and transportation planners can utilize these insights to make informed decisions about route expansion and fleet allocation, ensuring their operations align with demand patterns and maximize connectivity. Moreover, integrating network measures into demand forecasting models can significantly improve the accuracy of predictions, aiding airlines, governments, and other stakeholders in planning for the future. This, in turn, could lead to more targeted infrastructure investments in airports that serve as key connectivity hubs. Additionally, airlines can use network analysis to guide marketing strategies and the development of new routes, ensuring they effectively cater to the evolving demand for air travel.

## Conclusion

In conclusion, this study has demonstrated the potential of using network measures, particularly PageRank, to model and predict air travel demand by addressing the research question of how the global flight network structure impacts air travel patterns. The analysis highlights the importance of the number of airlines serving a route in influencing passenger behaviour, and the relevance of codeshare airlines in reflecting true travel demand. The analysis also suggests that passengers seem to be less influenced by the travel distance to the destination in making their travel. Moreover, while the influence of socio-economic factors such as GDP and the population is undeniable, network measures could complement traditional approaches based on population and GDP forecast to provide more accurate predictions of passenger volume.

The study underscores the value of network analysis in assessing airport passenger volume with respect to connectivity. It reveals that many Asian airports have higher-than-expected travel demand while European and North American airports tend to have lower demand, probably influenced by socio-economic factors like GDP and population as well as the availability of alternative modes of transport. Furthermore, regression models demonstrate that network measures can significantly enhance passenger volume prediction at both metropolitan and country levels. Importantly, network analysis of airports requires minimal detailed information, making it a computationally effective alternative to other data-intensive approaches such as machine learning. These findings have practical implications, aiding decision-making in aviation, such as predicting passenger volume for new flight routes based on network topology. Future research could explore the use of network measures in airport infrastructure planning and airline route optimization, evaluating airport and route performance based on network structure.
